# Raman Spectroscopic Imaging of the Whole *Ciona intestinalis* Embryo during Development

**DOI:** 10.1371/journal.pone.0071739

**Published:** 2013-08-20

**Authors:** Mitsuru J. Nakamura, Kohji Hotta, Kotaro Oka

**Affiliations:** Department of Biosciences and Informatics, Faculty of Science and Technology, Keio University, Kohoku-ku, Yokohama, Kanagawa, Japan; Academia Sinica, Taiwan

## Abstract

Intracellular composition and the distribution of bio-molecules play central roles in the specification of cell fates and morphogenesis during embryogenesis. Consequently, investigation of changes in the expression and distribution of bio-molecules, especially mRNAs and proteins, is an important challenge in developmental biology. Raman spectroscopic imaging, a non-invasive and label-free technique, allows simultaneous imaging of the intracellular composition and distribution of multiple bio-molecules. In this study, we explored the application of Raman spectroscopic imaging in the whole *Ciona intestinalis* embryo during development. Analysis of Raman spectra scattered from *C. intestinalis* embryos revealed a number of localized patterns of high Raman intensity within the embryo. Based on the observed distribution of bio-molecules, we succeeded in identifying the location and structure of differentiated muscle and endoderm within the whole embryo, up to the tailbud stage, in a label-free manner. Furthermore, during cell differentiation, we detected significant differences in cell state between muscle/endoderm daughter cells and daughter cells with other fates that had divided from the same mother cells; this was achieved by focusing on the Raman intensity of single Raman bands at 1002 or 1526 cm^−1^, respectively. This study reports the first application of Raman spectroscopic imaging to the study of identifying and characterizing differentiating tissues in a whole chordate embryo. Our results suggest that Raman spectroscopic imaging is a feasible label-free technique for investigating the developmental process of the whole embryo of *C. intestinalis*.

## Introduction

During embryogenesis, the intracellular composition and distribution of molecules control developmental processes, such as cell division, induction of cell differentiation, and morphological changes. Consequently, detecting and tracing the distribution of bio-molecules, such as mRNAs and proteins, in the developing embryo is one of the major challenges in developmental biology. Raman spectroscopic imaging is a useful technique for biological study [1] [2] [3]. Raman spectroscopy is a promising method for investigating the distribution of bio-molecules because it is a label-free and non- invasive technique. This unique combination of features allows simultaneous monitoring of intracellular composition and distribution of a number of bio-molecules that correspond to several kinds of cell activities, for example apoptosis and cell cycle [4] [5]. Therefore, Raman spectroscopic imaging can potentially be applied to acquisition of spatiotemporal information about the bio-molecules responsible for control of developmental processes.

Raman spectroscopy has been used as a label-free technique to distinguish anatomical features within the embryo [6] [7]. This method makes it possible to observe target tissues during embryogenesis without experimental difficulties such as injections, prior knowledge about target molecules to enable tissue-specific labeling, or preparation of fluorescent substances. In *Drosophila* and nematodes, major model organisms for developmental biology, different tissues or anatomical structures have been identified in a label-free manner using Raman spectroscopy, based on high-resolution detection of the distribution of biological molecules [6] [7] [8]. However, it is not known whether Raman spectroscopy can be used to identify tissues within the whole embryo during development.

Raman spectroscopy has also been widely known for its non-invasive and label-free method to detect and identify cell state [9] [10] [11]. By applying Raman spectroscopy to the developing embryo, it might be possible to visualize differences in cell state between blastomeres during cell differentiation by focusing on the spectral differences between cells. Differentiation of cultivated stem cells has been characterized using Raman spectroscopy, based on changes in intracellular biochemical composition [9] [10] [11]. Cell interactions are responsible for the fate determination of blastomeres in multicellular organisms [12] [13]. To date, however, no reports have described application of Raman spectroscopic imaging to the identification of differences in daughter cells dividing from the same mother cell in multicellular organisms.

In this study, we explored the application of Raman spectroscopic imaging during development of a chordate, *Ciona intestinalis*. This organism is well known and anatomically simple, with transparent embryos and appropriate embryonic size for imaging study (body length, ∼200 μm) [14]. In addition, another advantage of using *C. intestinalis* for developmental study is that it exhibits common features of the chordate body plan, and developmental staging has been well defined in each tissue, based on embryonic morphology [15]. Because *C. intestinalis* undergoes mosaic development, cell lineages are invariant among individuals [16] [17] [18] [19]. Furthermore, the cell-differentiation process of ascidians has been well studied, and the cell lineages have been completely described at single-cell resolution [20] [21] [22]. Here we demonstrate the application of Raman spectroscopic imaging to *C. intestinalis* embryogenesis, at the whole-body level, for the purpose of identifying and characterizing differentiating tissues.

## Materials and Methods

### Collection and preparation of *C. intestinalis* embryos

**Figure 1:**
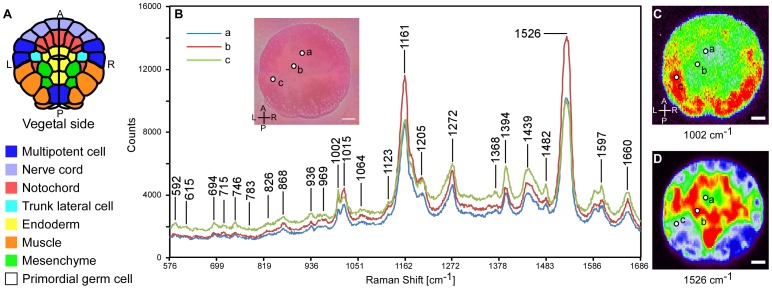
Raman spectra obtained from *C. intestinalis* embryo at the 112-cell stage. (A) Diagram of the *C. intestinalis* embryo from vegetal view. Each color in the diagram represents a distinguishable cell fate. (B) Raman spectra from three different spots (a, b and c in the inset bright-field image) in a 112-cell stage *C. intestinalis* embryo stained with CellMask. Numbers indicate the Raman band positions. Raman maps in Fig. 2 were generated from the corresponding Raman bands indicated by each latter and band position. (C and D) Raman maps constructed from the intensities of the individual Raman bands in the 998–1007 (C) and 1499–1552 (D) cm^−1^ range. The colors depict the Raman intensity of a given spectrum: where red represents the maximum intensity and black represents zero intensity. Abbreviations: A, Anterior; P, Posterior; L, Left; R, Right. Scale bar: 20 μm.

Adult *Ciona intestinalis* were obtained from the Maizuru, Misaki (National Bio-Resource Project), or Yokohama region of Japan. *Ciona* samples were treated in accordance with guideline of the committee of animal experiment in Keio University. Permission number is 2072. No specific permits were required for the described field studies. Eggs and sperm were obtained surgically from the gonoduct. After dechorionation, eggs were inseminated and incubated at 18°C in the dark until the 2-, 4-, 8-, 16-, 32-, 64-, 112-cell, gastrula, neurula, and tailbud stage, determined according to standard criteria for *C. intestinalis* developmental stages [15]. Embryos at each stage were fixed at room temperature in 4% paraformaldehyde (Sigma-Aldrich) in MOPS buffer (pH 7.5) for 30 minutes, and then washed twice for 30 minutes in PBS. Though cell boundaries of transparent *C. intestinalis* embryos could be observed using bright-field and differential-interference microscopy, it was difficult to identify them in the bright-field images acquired by the Raman-microscopy technique used in this study (Fig. 1B). To overcome this difficulty, CellMask Orange Plasma Membrane Stain (Invitrogen) was used, solely to enable recognition of cell boundaries. Fixed embryos were stained at 18°C in 0.001% CellMask in PBS for 1 hour in the dark and washed once in PBS before Raman microscopic observation to visualize cell boundaries by Raman spectrum analysis. To inhibit muscle differentiation of A8.16 blastomere (secondary muscle), U0126 (Promega) was added to embryos at a concentration of 10 μmol/l at the 8-cell stage. Further, cleavage of embryos was permanently arrested by treatment with cytochalasin D (Tocris) at a concentration of 4 μg/ml at the 76-cell stage until fixation. Embryos were cultured until control embryos reached hatching stage (22 hpf), and fixed with paraformaldehyde by the method described above. In controls, embryos were treated with 0.1% DMSO (Nacalai Tesque), the solvent of U0126. Muscle cells in DMSO or U0126-treated embryos were histochemically stained for acetylcholinesterase (AChE) by the method described by Karnovsky and Roots [23] with acetylcholine iodide (Sigma-Aldrich) as substrate.

### Raman spectroscopy

It is difficult to perform time-lapse imaging on live embryos, in which internal states like molecular composition and localization are changing, because *C. intestinalis* develops too rapidly to allow collection of Raman signals sufficiently intense for real-time acquisition of Raman maps. Therefore, we investigated changes of internal states over a developmental time course by comparing fixed embryos whose developmental stages had been established according to morphological criteria. Several features of the *C. intestinalis* embryo, including its typical mosaic development, invariant cell lineages and cell position [16] [17] [18] [19], and defined developmental staging based on embryonic morphology [15], permitted tissue recognition in fixed embryos. Raman spectra of *C. intestinalis* embryos in PBS were obtained using an inVia confocal microscope system (Renishaw), using a 785-nm laser in streamline mode and a 63× water-immersion objective (Leica, NA 0.9). Laser power in the scanning line was 90 mW. Raman spectra were acquired from 577–1686 cm^−1^ using a 1200-lines/mm grating. The scanning step size was 1.2, 2.3, or 3.4 μm in both the x and y directions. Raman spectra in points along a single line were collected simultaneously. In each line, the signal was collected for 40 seconds. The scanning of one plane of an embryo was completed in just over 3 hours.

### Data analysis of Raman spectra and maps

Data collection, analysis of Raman spectra, and construction of Raman maps were carried out using the Renishaw WiRE software, version 3.3. Raman maps, gray scale and rainbow scale maps representing Raman intensity at each point on embryo, were acquired based on the evaluated spectral area for each Raman band. Gray scale maps representing Raman intensity as a gray value were used for analysis. Analysis of constructed Raman maps was performed using the open-source software Fiji [24]. For the first step of analysis, background (determined from the gray value of the PBS area) was subtracted. Then, the Raman intensities at each point on the embryo were measured and compared with the gray value. In comparisons of Raman intensity between muscle/endoderm cells and daughter cells with other fates, Student's t-test was performed to determine statistical significance.

## Results and Discussion

### Raman spectra of *C. intestinalis* embryo

Until the blastula stage, embryos of *Ciona intestinalis* have a two-layered structure consisting of animal and vegetal hemispheres. The vegetal hemisphere was chosen to monitor fixed embryos at the 112-cell stage because more cell fates were determined in the vegetal hemisphere than the animal hemisphere [22] (Fig. 1A).

Raman spectroscopy was applied to *C. intestinalis* embryos and spectra scattered from embryos were collected (Fig. 1B). According to the Raman spectrum, certain Raman bands were detected (Fig. 1B). Raman intensity was calculated based on the spectral area of each band, and Raman maps in a rainbow scale depicting Raman intensity at each spot on the embryo were constructed from the spectral dataset (Fig. 1C and 1D).

### Multiple localization patterns of Raman intensity within the *C. intestinalis* embryo can be identified in Raman maps

**Figure 2:**
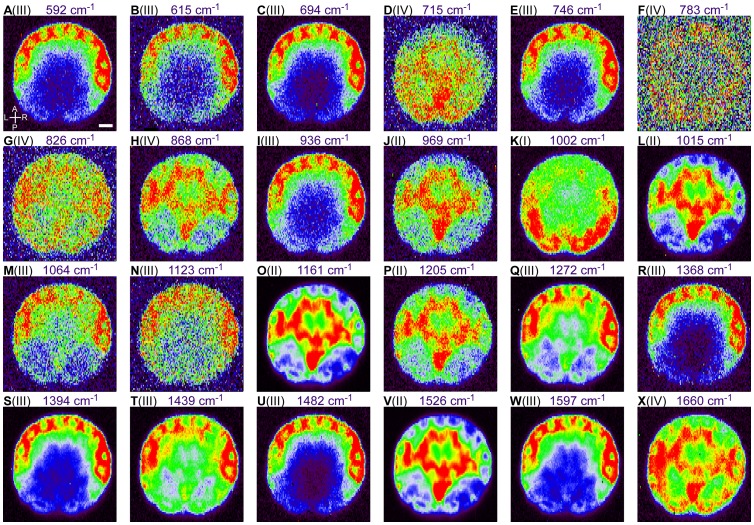
Raman maps generated from each individual Raman band of a 112-cell stage *C. intestinalis* embryo. (A–X) Image panels displaying Raman maps of the 112-cell stage *C. intestinalis* embryo stained with CellMask in a rainbow scale with red representing the highest intensity and black representing the lowest. These maps were constructed based on the intensity of each individual Raman band indicated above. Maps were classified into four groups (I–IV) as described in the Results and Discussion. Roman numerals (I–IV) represent the group into which each Raman map was classified. Abbreviations: A, Anterior; P, Posterior; L, Left; R, Right. Scale bar: 20 μm.

To visualize the distribution of molecules during *C. intestinalis* embryogenesis, Raman maps for each individual Raman band (Fig. 1B and Table 1) at the 112-cell stage were constructed at 1.2×2.3-μm resolution (Fig. 2). After spectral analysis, a number of localized distribution patterns of high–Raman intensity spots were detected. Raman maps constructed from low-intensity Raman bands yielded noisy images (e.g. 826 cm^−1^ in Fig. 1B and Fig. 2G). The resulting Raman bands and maps were classified into four groups (I–IV) according to the distribution pattern of the intensity (Fig. 2 and Table 1). In group I, Raman maps were constructed from the Raman band located at 998–1007 cm^−1^, and high intensity was detected at muscle cells [22] (Fig. 1A, 2K and Table 1). In group II, high intensity was detected in the endoderm and notochord cell regions [22] (Fig. 1A, 2 and Table 1). Maps of group III were constructed from the CellMask Raman band (Fig. S1 and Table 1). Localized distribution of high-intensity spots effectively depicted the cell shape of each blastomere in the peripheral region of the 112-cell stage embryo (Fig. 2). Raman maps of group IV exhibited dissimilar distribution from those of the other three groups in embryos at the 112-cell stage (Fig. 2 and Table 1) and other stages. Almost all of the group-IV maps were strongly affected by background noise (Fig. 2D, 2F, 2G, and 2H). The distribution pattern of Raman maps derived from the Raman band at 868 cm^−1^ was similar to that of group-II maps at the 112-cell stage (Fig. 2H), but had little in common with embryos at other stages. For this reason, the Raman map for 868 cm^−1^ was categorized into group IV.

**Table 1:** Band positions and tentative assignments of major Raman bands detected from *C. intestinalis* embryo.

	Signal to baseline				
in Fig. 2	1st [cm^−1^]	2nd [cm^−1^]	Position [cm^−1^]	group	Assignments
A	578	600	592	III	CellMask
B	600	633	615	III	CellMask
C	677	707	694	III	CellMask
D	707	727	715	IV	Choline (H_3_C)N+, mitochondria
E	734	762	746	III	CellMask
F	776	790	783	IV	cytosine, urasil
G	812	841	826	IV	Tyr, ν(O-P-O), DNA
H	856	882	868	IV	Trp, Tyr
I	925	946	936	III	CellMask, ν(C-C), (C-O-C), glycogen
J	948	981	969	II	δ( = C-H), (C-C) backbone, retinoids
K	998	1007	1002	I	Phe, myoplasm, mitochondoria
L	1008	1026	1015	II	carotenoids, retinoids
M	1047	1073	1064	III	CellMask, Chain C-C
N	1116	1129	1123	III	CellMask, ν(C-C), (C-N)protein, ν(C-O)
O	1134	1175	1161	II	myoplasm, carotenoids, retinoids
P	1191	1214	1205	II	Tyr, Phe, retinoids
Q	1241	1288	1272	III	CellMask, δ( = C-H), Trp, ν(CN), δ(NH)
R	1350	1380	1368	III	CellMask, globin
S	1382	1410	1394	III	CellMask, CH rocking
T	1427	1469	1439	III	CellMask, CH_2_ deformation, myoplasm, mitochondoria, retinoids
U	1475	1489	1482	III	CellMask
V	1499	1552	1526	II	myoplasm, carotenoids
W	1572	1625	1597	III	CellMask, retinoids, nucleic acids
X	1644	1676	1660	IV	ν(C = C) lipid, myoplasm, mitochondoria

Assignments were compiled from previous articles [8], [28], [31]–[32], and [37]–[40].

The distribution of Raman intensity of group-III maps with CellMask stained embryos varied greatly from that with CellMask-free embryos (Fig. 2 and S2). This is due to the strong signals represented by CellMask (Fig. S1), and Raman maps of group III were strongly affected as a result (Fig. 2 and S2). On the other hand, distributions of intensity categorized into groups I, II, and IV were maintained in the absence of CellMask staining (Fig. 2 and S2). High Raman-intensity spots in maps of groups I and II closely corresponded with muscle and endoderm regions in the absence of CellMask (Fig. S2).

### Muscle and endoderm in the whole embryo can be identified with Raman spectroscopy in a label-free manner by focusing on molecular distribution

**Figure 3:**
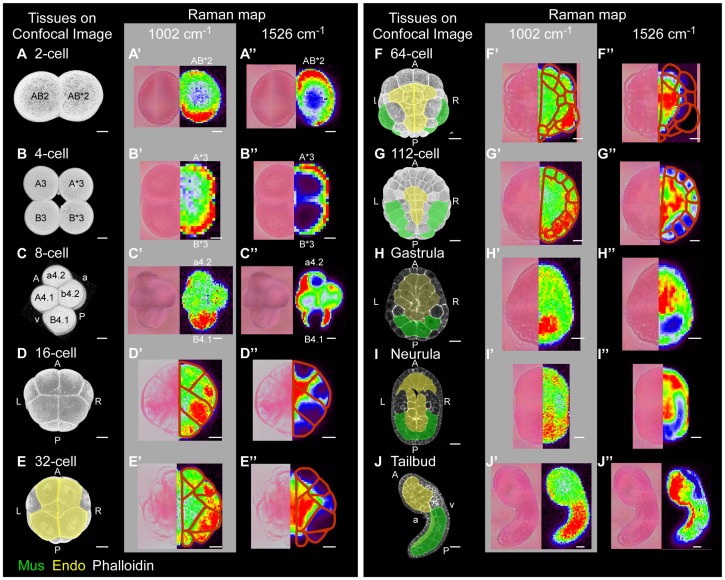
Raman maps of *C. intestinalis* embryos from 2-cell to tailbud stage. (A–J) Confocal 3D (from 2-cell to 112-cell stages) and section images from dorsal (gastrula and neurula stages) or side views (tailbud stage) of *C. intestinalis* embryos stained with phalloidin (white). Images show the location of differentiated muscle (green) and endoderm (yellow) within the embryo. (A’ –J’ and A’’ –J’’) Bright-field (left) and Raman maps in a rainbow scale (right) constructed from the intensity of single bands at the 1002 (A’ –J’) and 1526 (A’’ –J’’) cm^−1^ at each stage. Because the *C. intestinalis* embryo is bilaterally symmetrical, Raman maps of only half of the embryo are represented. Red line in maps from 16-cell to 112-cell stage represents the presumed cell border. To identify the cell boundary, the bright-field images and Raman maps constructed from Raman bands of CellMask (group III) were used. In addition to the Raman band at 1526 cm^−1^, Raman maps constructed from other bands of group II (Fig. 2 and Table 1) also represented similar distribution of high-intensity spots (see Fig. S3). Abbreviations: A, Anterior; P, Posterior; L, Left; R, Right; a, animal; v, vegetal; Mus, muscle; Endo, endoderm. Scale bar: 20 μm.

We also used Raman spectroscopy to investigate *C. intestinalis* embryos from 2-cell to tailbud stage (Fig. 3). We focused on Raman bands of groups I and II, because the distribution of high-intensity spots in these maps corresponded closely to muscle and endoderm/notochord, respectively, in the 112-cell stage embryo (Fig. 2). Raman maps were compared with anatomical information about muscle and endoderm at each developmental stage [15] (Fig. 3A–3J and S3).

Before the 16-cell stage, the cell fates of all blastomeres are not specified. At the 2-cell and 4-cell stages, high–Raman intensity spots were concentrated into the posterior sides of group-I Raman maps (Fig. 3A’ and 3B’), whereas group-II Raman bands exhibited higher intensity on the anterior side of the embryo than the posterior side (Fig. 3A’’ and 3B’’). At the 8-cell stage, high intensity was observed in B4.1 cells in group-I Raman maps (Fig. 3C’). In group-II maps, high-intensity spots corresponded to the cell membrane in 8-cell embryos (Fig. 3C’’). At the 16-cell stage, localization of high-intensity spots within cells in Raman maps of groups I and II was seen, but the distribution patterns of these two groups were different (Fig. 3D’ and 3D’’).

Muscle and endoderm cells differentiate after the 64-cell and 32-cell stages, respectively [22] (Fig. 3E–3J). High–Raman intensity region in Raman maps of groups I and II were consistent with the locations and structures of differentiated muscle and endoderm, respectively, both in the presence (Fig. 3 and S3, red) and absence (Fig. S2 and S4, red) of CellMask. In our study, muscle and endoderm within the whole *C. intestinalis* embryo could be respectively identified in all of the developmental stages we investigated, by taking note of the distribution of bio-molecules of groups I and II as detected by Raman spectroscopy. As noted above, no label was necessary.

#### Assignment of Raman band of group I

Detection of a strong Raman signal at the location of muscle in group-I maps (Fig. 3F’–3J’) indicated that muscle cells possess higher levels of certain molecular species than other tissues, which have major effects on the group-I Raman maps. Distribution patterns of myoplasm, which is abundant in muscle-lineage blastomeres of ascidians [25] [26] [27], seemed to closely coincide with the distribution patterns of strong signals in group-I Raman maps from the 2-cell to tailbud stage (Fig. 3A’–3J’, red). Raman spectra of myoplasm exhibit strong signals at 1003, 1158, 1449, 1521, and 1645 cm^−1^ [28], respectively corresponding to Raman bands at 1002, 1161, 1439, 1526, and 1660 cm^−1^ (Table 1) in our data. In fact, Raman maps constructed from the Raman bands at 1002, 1439, and 1660 cm^−1^ exhibited strong signals in regions containing myoplasm (muscle) in tailbud-stage embryos under CellMask-free conditions (Fig. S4A, red). However, Raman maps constructed from the bands at 1161 and 1526 cm^−1^ did not exhibit strong signal at muscle (Fig. S4A, red). Instead of or in addition to muscle, Raman maps of 1161, 1439, 1526, and 1660 cm^−1^ showed high–Raman intensity spots in endoderm regions (Fig. S4A, red). Based on the positions of the Raman bands, it is possible that high intensities of other molecules such as lipids, carotenoids, and retinoids could strongly affect these Raman maps (Fig. S1, S4 and Table 1).

In addition to myoplasm, the distribution of strong signal in Raman maps of group I (Fig. 3A’–3J’) seemed to be similar to the reported distribution of mitochondria [29] [30]. Indeed, Raman maps constructed from Raman bands at 715, 1002, 1439, and 1660 cm^−1^, which are major bands of mitochondria [31] [32], also exhibited strong Raman signal at muscle regions under CellMask-free conditions (Fig. S4B). The Raman band at 1002 cm^−1^ is assigned to phenylalanine and is a typical proteins band [28]. Therefore, myoplasm or mitochondria, or proteins co-localized with them, are potential sources of major effects on Raman maps of group I.

#### Assignment of Raman bands of group II

Whereas distribution of high-intensity regions in Raman maps from the 2-cell to 8-cell stage did not coincide with the reported distribution of yolk granules, distribution of strong signals in group-II Raman maps from the 16-cell to tailbud stage seemed to closely coincide with the distribution patterns of the yolk granules, which are abundant in endoderm blastomeres of ascidians [27] (Fig. S3, red). Yolks of oviparous vertebrates store carotenoids [33] [34] [35], and storage of carotenoids in ascidian eggs has also been demonstrated [36]. Carotenoids have strong Raman bands at approximately 1010, 1155, and 1525 cm^−1^ [37] [38]. Our results reveal that the distribution of high-intensity spots in Raman maps from the 16-cell to tailbud stage, constructed from the corresponding Raman bands at 1015, 1161, and 1526 cm^−1^, classified into group II, closely coincided with the reported distribution of yolk granules (endoderm) [27] (Fig. S4C). In addition to carotenoids, Raman bands at 969, 1015, 1161, 1205, 1439, and 1597 cm^−1^ [39] [40], major bands of retinoids co-localized with yolk [41], also generated Raman maps that exhibited strong signals at endoderm regions under CellMask-free conditions (Fig. S4D). In the Raman map of 1439 cm^−1^, Raman bands of mitochondria and myoplasm as well as retinoids might affect the distribution of high-intensity regions. Carotenoids and retinoids co-localized with yolk granules are plausible candidates for the major contributors to the Raman bands of group II. Because notochord, as well as endoderm, contains yolk granules [27], we also detected high Raman intensity at the location of the notochord in the 112-cell stage embryo (Fig. 3G’’).

### Simultaneous detection of molecular distributions within the *C. intestinalis* embryo during the cell-differentiation process

Next, we investigated the relationship between Raman-intensity distribution and cell-differentiation processes of muscle and endoderm in the whole *C. intestinalis* embryo. Raman maps of groups I (1002 cm^−1^) and II (1526 cm^−1^) were acquired from embryos at the 16-cell, 32-cell and 64-cell stage, when cell differentiation of these two tissues occurs [22].

**Figure 4:**
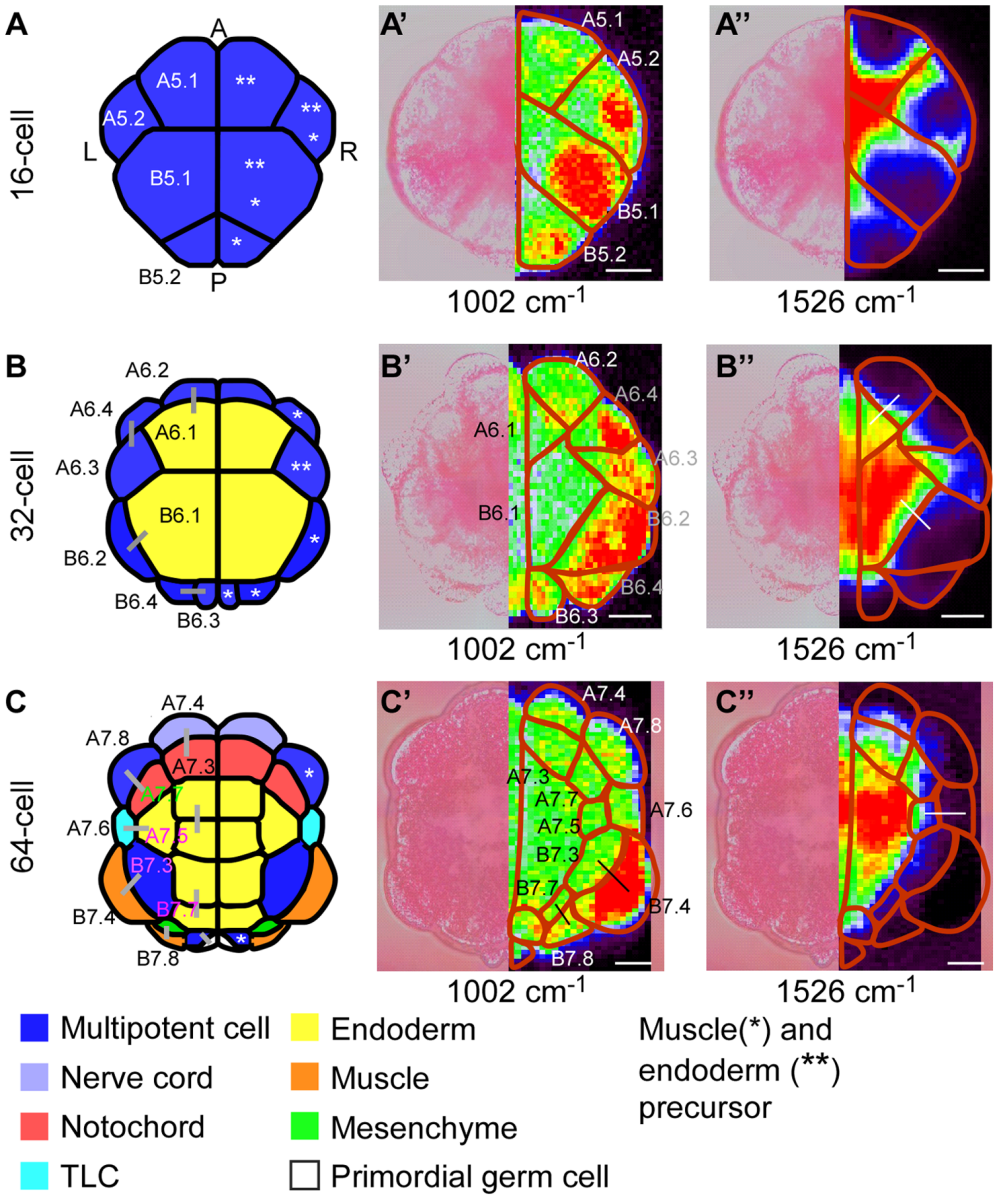
Localized distribution of Raman intensity during cell differentiation process. Vegetal views of the embryo at each stage. 16-cell (A–A’’). 32-cell (B–B’’) and 64-cell (C–C’’). (A–C) Diagrams with cell lineage and fate information. Cell fates of each blastomere are indicated by different colors in the diagrams. Pairs of daughter cells are indicated by gray lines. (A’ –C’ and A’’ –C’’) Raman maps generated from the intensity of single Raman bands at 1002 (A’ –C’) and 1526 (A’’ –C’’) cm^−1^. Bright-field images (left) are represented with Raman maps (right). A portion of presumed cell border is indicated by red line. This cell border was determined from the bright-field images and Raman maps of group III. Daughter cell pairs in which, one daughter produced differentiated muscle (black line, C’)/endoderm (white line, B’’ and C’’) and another daughter produced other fate cells are indicated by each line within the embryo. Abbreviations: A, Anterior; L, Left; P, Posterior; R, Right; Scale bar. 20 μm.

High–Raman intensity spots of groups I (1002 cm^−1^) and II (1526 cm^−1^) were respectively seen in muscle and endoderm precursors, as well as differentiated muscle and endoderm blastomeres, in contrast to blastomeres with other fates (Fig. 4A’–4C’ and 4A’’–4C’’). Furthermore, we observed localization of high–Raman intensity spots within the mother cells of muscle/endoderm lineage cells (Fig. 4, asterisks: A5.2, B5.1, B5.2, and A6.4 for muscle mother cells; A5.1, A5.2, B5.1, and A6.3 for endoderm mother cells). Distribution patterns of high–Raman intensity spots between groups I and II differed both in A5.2 and B5.1 mother cells, even though they were observed at the same time point (Fig. 4A’ and 4A’’). These results suggest that distribution of molecules were controlled in specific regions, in which each type of molecules was concentrated within mother cells before cell division.

#### Muscle/endoderm daughter cells exhibit higher Raman intensity than daughter cells that differentiate into other tissues

**Figure 5:**
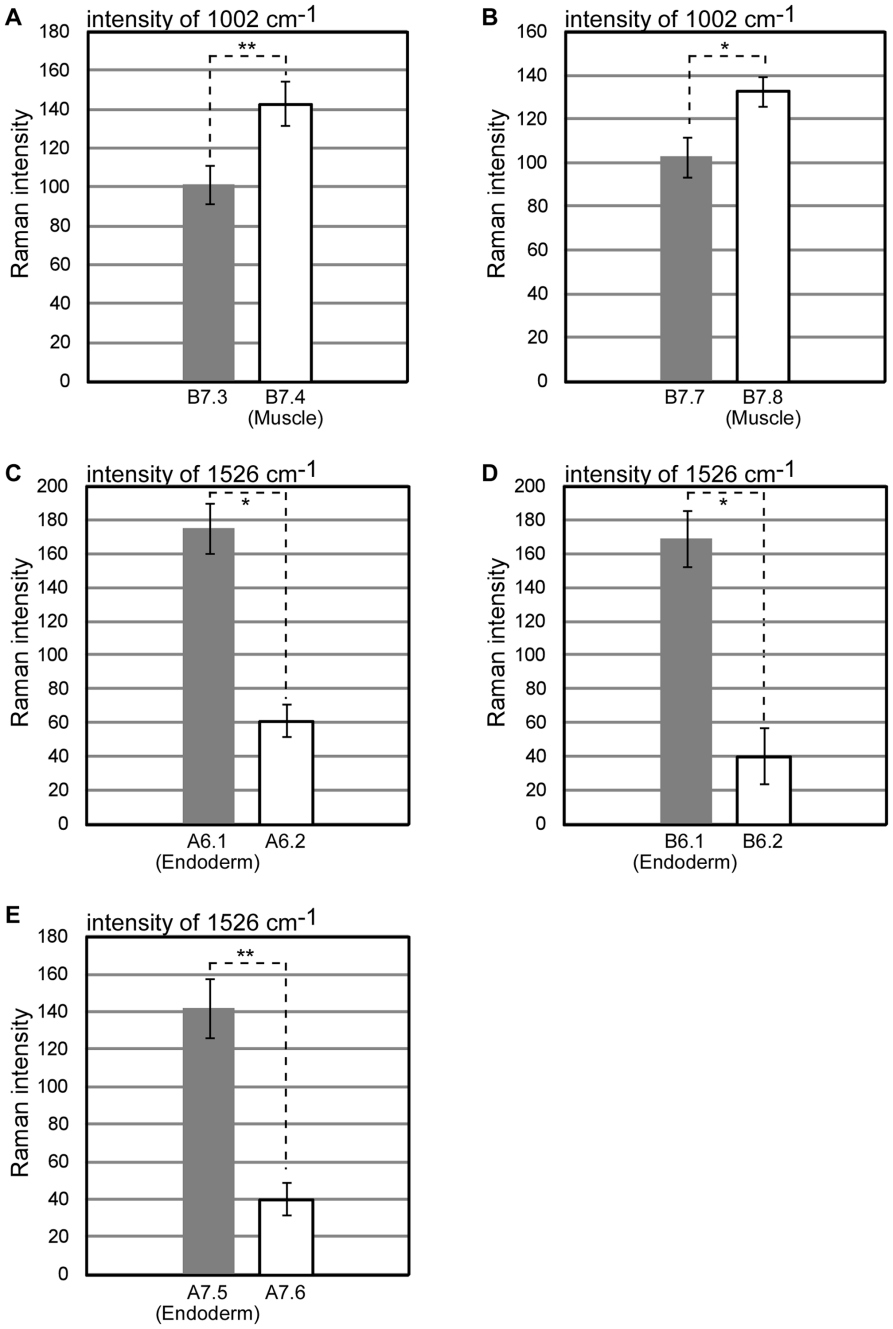
Quantitative difference in Raman intensity between differentiated muscle/endoderm and daughter cells with other fates. (A–E) Raman intensities of A6.1, A6.2, B6.1, and B6.2 blastomeres, were acquired from 32-cell stage embryo (four individuals), and intensities of A7.5, A7.6, B7.3, B7.4, B7.7 and B7.8 blastomeres were acquired from 64-cell stage embryos (five individuals). Cell fate and embryonic location of each blastomere are shown in Fig. 4. Student's t-test results show significant differences for the intensities of Raman bands at 1002 (A and B) and 1526 (C–E) cm^−1^ in Raman spectra of differentiated muscle/endoderm and other fate daughter cells (*p<0.005, **p<0.001). Error bars represent the standard error of the mean (SEM).

We observed a major gap in Raman intensities between pairs of daughter cells; one differentiated into muscle/endoderm blastomere while the other differentiated into a blastomere with different fate (for muscle, two pairs of black lines in Fig. 4C’, 5A, and 5B; for endoderm, three pairs of white lines in Fig. 4B’’, 4C’’, and 5C–5E). In group I, Raman intensities of daughter cells that differentiated into muscle were significantly higher than those of daughter cells that formed other tissue types (Fig. 4C’, 5A, and 5B). Similarly, in group II, Raman intensities of daughter cells that produced endoderm were significantly higher than those of daughter cells with different fate (Fig. 4B’’, 4C’’, and 5C–5E). Thus, after division from the same multipotent mother cells in a whole chordate embryo, we succeeded in effectively distinguishing differentiated muscle/endoderm daughter cells from daughter cells with other fates based on the significant differences in Raman intensity.

### Raman spectroscopic imaging as a useful tool for developmental study

Raman spectroscopic imaging permits label-free examination of anatomical structures and tissue development. In the case of *Drosophila*, Raman spectroscopic imaging has been employed to monitor the fat-body remodeling at the pupal stage during metamorphosis. Lipid droplets in the fat body and pupal fat cells have been observed by CARS/TPE-F microscopy to achieve label-free imaging [7]. In the case of nematodes, fine details within the body have been distinguished using information about the spatial distribution of bio-molecules, obtained by FPA-FTIR and Raman spectroscopic imaging [8]. To date, however, no report has described identification of tissues within a whole embryo during the process of development. In our study, Raman spectroscopic imaging made it possible to distinguish muscle and endoderm during development of the *C. intestinalis* embryo, just after the differentiation of these two tissues took place (Fig. 3). For an application of our study result, inhibition of muscle differentiation of A8.16 blastomere in U0126-treated embryos was identified with Raman spectroscopy by focusing on the intensity of 1002 cm^−1^ band (Fig. S5D). This result shows that muscle lineage perturbation also can be detected through Raman spectra.

The cell state of cultivated stem cells can be distinguished by focusing on changes in the Raman spectrum during development of these cells [9] [10] [11]. In these previous studies, differentiation of embryonic stem (ES) cells was triggered in aggregates of ES cells termed embryoid bodies. Because cell interactions are responsible for fate determination of blastomeres in multicellular organisms [12] [13], identification of cell state in the context of embryogenesis at the whole-body level will promote greater understanding of cell-differentiation processes. In our application of Raman spectroscopic imaging to whole embryos of *C. intestinalis*, quantitative comparison of Raman intensity allowed us to effectively distinguish between muscle/endoderm cells and daughter cells with other fates that had divided from the same multipotent mother cells (Fig. 4 and 5).

### Summary

We applied Raman spectroscopy as a label-free imaging technique for studying the developmental process of *Ciona intestinalis*. We observed whole embryos, from the 2-cell to tailbud stage, and investigated whether Raman spectroscopic imaging can distinguish tissues within the whole embryo in a label-free manner. In addition to the label-free identification, another advantage of the Raman spectroscopic method is the simultaneous speculation of multiple molecules, including molecules difficult to label, localized in each tissue. In this report, the information of different tissue-specific bio-molecules could be acquired in a single shot. The detected localized distribution of Raman intensity made it possible to identify muscle and endoderm within whole embryos at multiple stages, just after the cell fates of these tissues had been determined. In addition, the intracellular distribution of bio-molecules during the cell-differentiation process from the 16-cell to 64-cell stage was investigated using the obtained Raman maps. The Raman intensity of the individual Raman bands at 1002 and 1526 cm^−1^ differed significantly between muscle/endoderm cells and daughter cells with other fates that had divided from the same mother cells. These results suggest that Raman spectroscopy can be used to effectively distinguish cell fates in daughter cells, for the purposes of study of the cell-differentiation process, in whole embryos. Thus, our results demonstrate that Raman spectroscopic imaging is a useful label-free technique for investigations of developmental biology.

## Supporting Information

Figure S1 **Comparison of Raman spectra with or without CellMask staining at the 112-cell stage.** Raman spectra acquired from *C. intestinalis* embryo at the 112-cell stage with (red) and without (blue) CellMask staining are shown. Raman maps in Fig. S2 were generated from the corresponding Raman bands indicated by each letter and band position. Roman numerals (I–IV) represent the groups into which each Raman band was classified.
(TIF)

Figure S2 **Raman maps generated from each individual Raman band in 112-cell stage embryos in CellMask-free condition.** Image panels displaying Raman maps of the 112-cell stage *C. intestinalis* embryo in the absence of CellMask in a rainbow scale with red representing the highest intensity and black representing the lowest. These maps were constructed based on the intensity of each individual Raman band indicated to the left. Roman numerals (I–IV) represent the group into which each Raman map was classified. Abbreviations: A, Anterior; P, Posterior; L, Left; R, Right. Scale bar: 20 μm.
(TIF)

Figure S3 **Raman maps of group II from 2-cell to tailbud stage embryos stained with CellMask.** (A–J) (left) Confocal 3D (from 2-cell to 112-cell stages) and section images from dorsal (gastrula and neurula stages) or side views (tailbud stage) of *C. intestinalis* embryos stained with phalloidin (white). Images show the location of differentiated muscle (green) and endoderm (yellow) within the embryo. Right halves of Raman maps in a rainbow scale constructed from Raman bands at 969, 1015, 1161, 1205, and 1526 cm^−1^ are represented with bright-field image (left half) at each stage. These bands were categorized into group II according to the standard written in Results and Discussion. Embryos were stained with CellMask. Red line in Raman maps from 16-cell to 112-cell stage represents cell border presumed from the bright-field images and Raman maps of group III. Abbreviations: A, Anterior; P, Posterior; L, Left; R, Right; a, animal; v, vegetal; Mus, muscle; Endo, endoderm. Scale bar: 20 μm.
(TIF)

Figure S4 **Raman maps constructed from major Raman bands of myoplasm, mitochondria, carotenoids and retinoids.** Raman maps constructed from *C. intestinalis* embryos at the tailbud stage in the absence of CellMask. (A–D) Raman maps that represent Raman intensity at each embryonic region, depicted in rainbow scale. These maps were constructed from major Raman bands of myoplasm (A), mitochondria (B), carotenoids (C), and retinoids (D). Roman numerals (I–IV) represent the group into which each Raman band was classified. (Right panel) Confocal section image from side views of *C. intestinalis* embryo at the tailbud stage stained with phalloidin (white). Image shows the location of differentiated muscle (green) and endoderm (yellow) within the embryo. Abbreviations: A, Anterior; P, Posterior; a, animal; v, vegetal; Mus, muscle; Endo, endoderm. Scale bar: 20 μm.
(TIF)

Figure S5 **Verification of muscle differentiation of A8.16 blastomere by acetylcholinesterase (AChE) activity and Raman spectroscopy.** (A–D) Muscle differentiation was verified by histochemical staining for AChE activity (A and B, brown) and Raman spectroscopy (C and D, red). Raman maps (C and D) were constructed based on the intensity of the single Raman band at the 1002 cm^−1^, and were represented in a rainbow scale. The scanning step size was 5.6 μm in both the x and y directions. Embryos were treated with DMSO (A and C) or U0126 (B and D) at the 8-cell stage, cytochalasin D at the 76-cell stage, and cultured until hatching stage (22 hpf). Arrowheads indicate A8.16 blastomeres. Inhibition of muscle differentiation of A8.16 blastomeres in U0126-treated embryos was detected by histochemical staining for AChE activity because brown products were not observed in A8.16 blastomeres (B, arrowheads). (C and D) Dotted white lines show region of interests (ROIs) encompassing A8.16 blastomere (ROI 1 and 3) and primary muscle region (ROI 2 and 4). Each ROI was used for the calculation of the intensity. Student's t-test result showed significant differences between the intensity of A8.16 blastomere and primary muscle region in U0126-treated embryo (n = 6, p<0.005) while DMSO-treated embryo showed no significant differences (n = 4, p>0.15). All embryos are in vegetal pole view. Abbreviations: A, Anterior; P, Posterior; L, Left; R, Right. Scale bar: 20 μm.
(TIF)
