# Supplementary figures and images for: Raman Spectroscopic Imaging of the Whole Ciona intestinalis Embryo during Development
Source: PLoS One. 2013 Aug 20;8(8):e71739. doi: 10.1371/journal.pone.0071739 (PMC3748111; doi:10.1371/journal.pone.0071739)

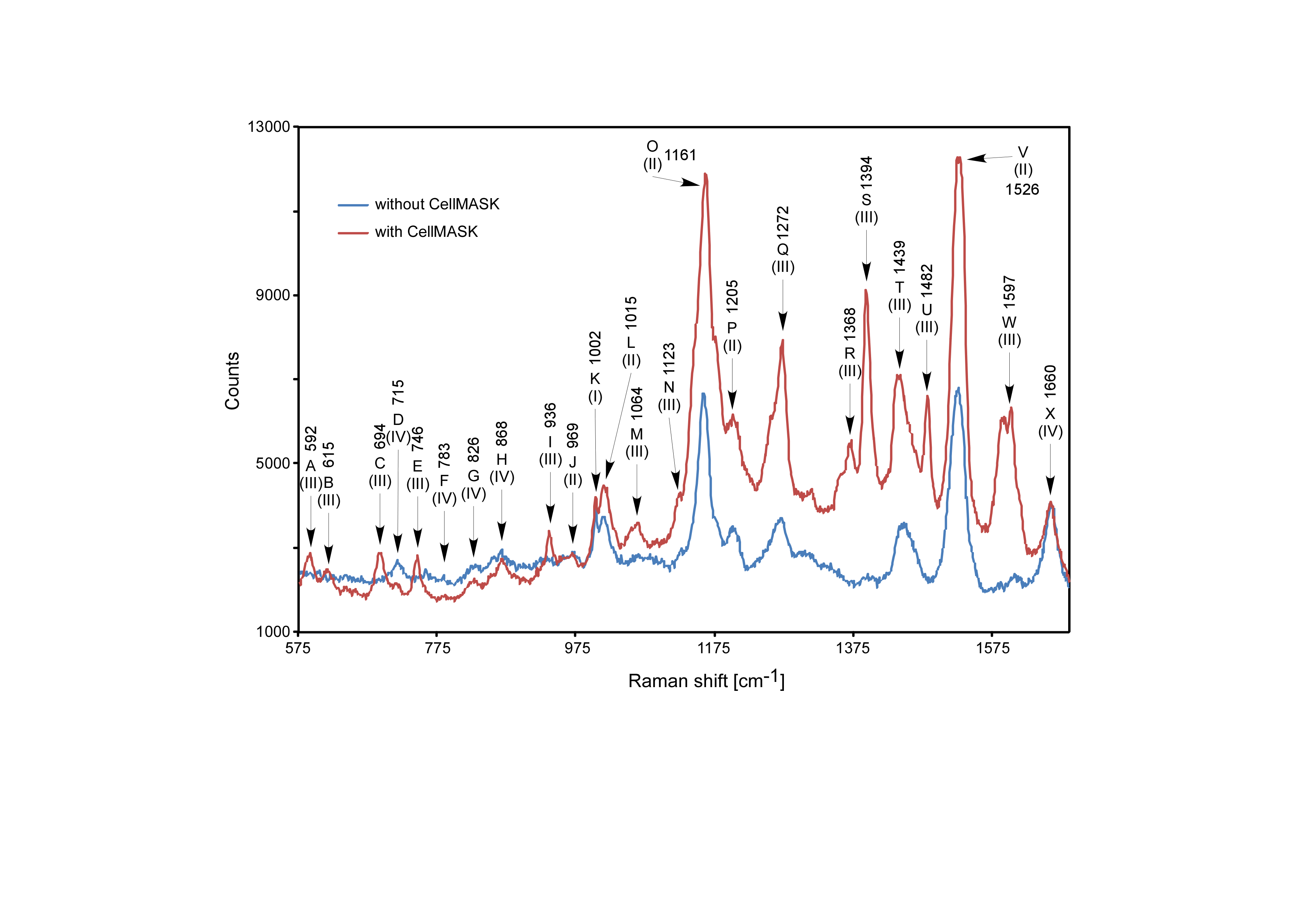

Supplement: Figure S1 — Comparison of Raman spectra with or without CellMask staining at the 112-cell stage. Raman spectra acquired from C. intestinalis embryo at the 112-cell stage with (red) and without (blue) CellMask staining are shown. Raman maps in Fig. S2 were generated from the corresponding Raman bands indicated by each letter and band position. Roman numerals (I–IV) represent the groups into which each Raman band was classified. (TIF) [file pone.0071739.s001.tif]

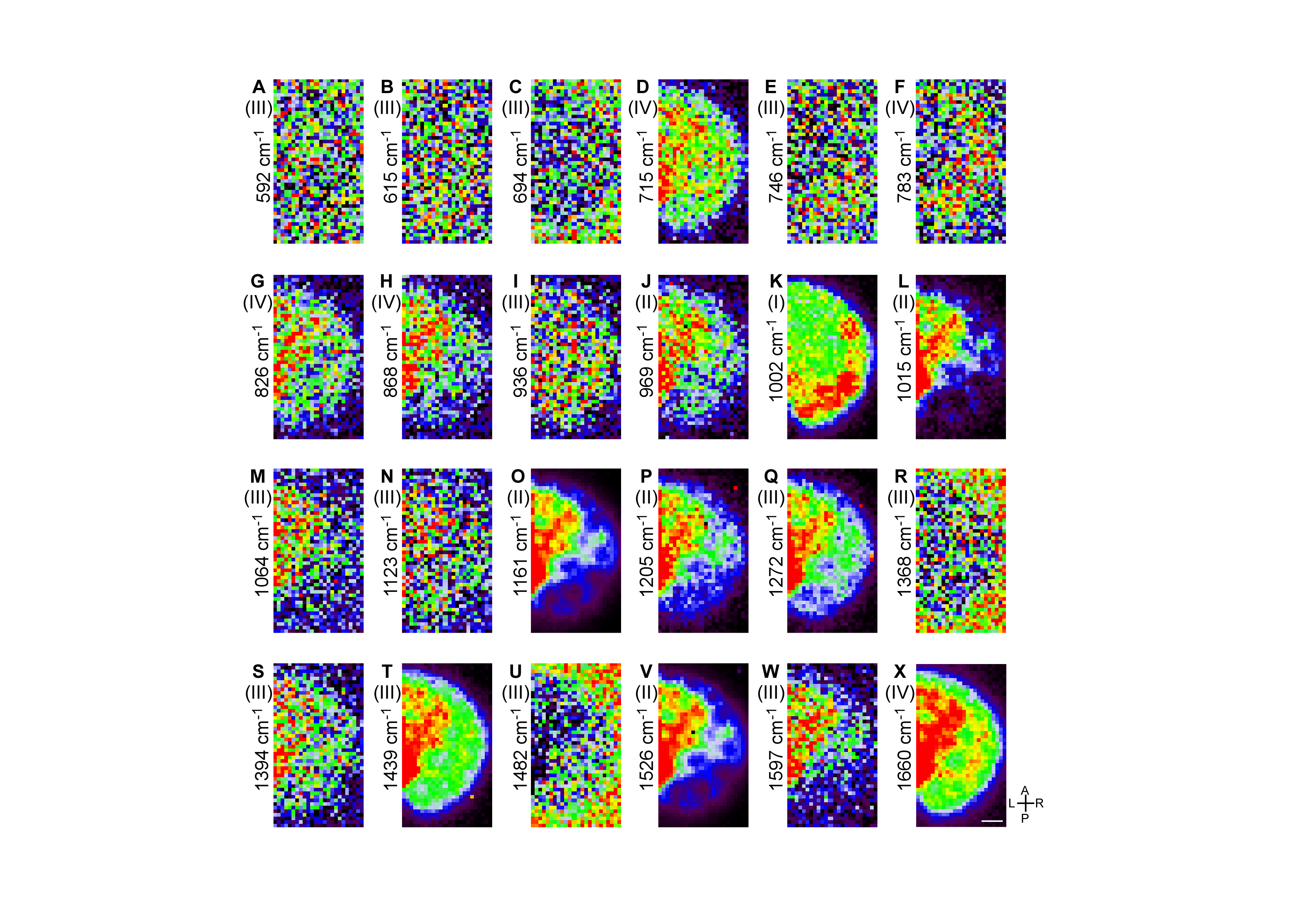

Supplement: Figure S2 — Raman maps generated from each individual Raman band in 112-cell stage embryos in CellMask-free condition. Image panels displaying Raman maps of the 112-cell stage C. intestinalis embryo in the absence of CellMask in a rainbow scale with red representing the highest intensity and black representing the lowest. These maps were constructed based on the intensity of each individual Raman band indicated to the left. Roman numerals (I–IV) represent the group into which each Raman map was classified. Abbreviations: A, Anterior; P, Posterior; L, Left; R, Right. Scale bar: 20 μm. (TIF) [file pone.0071739.s002.tif]

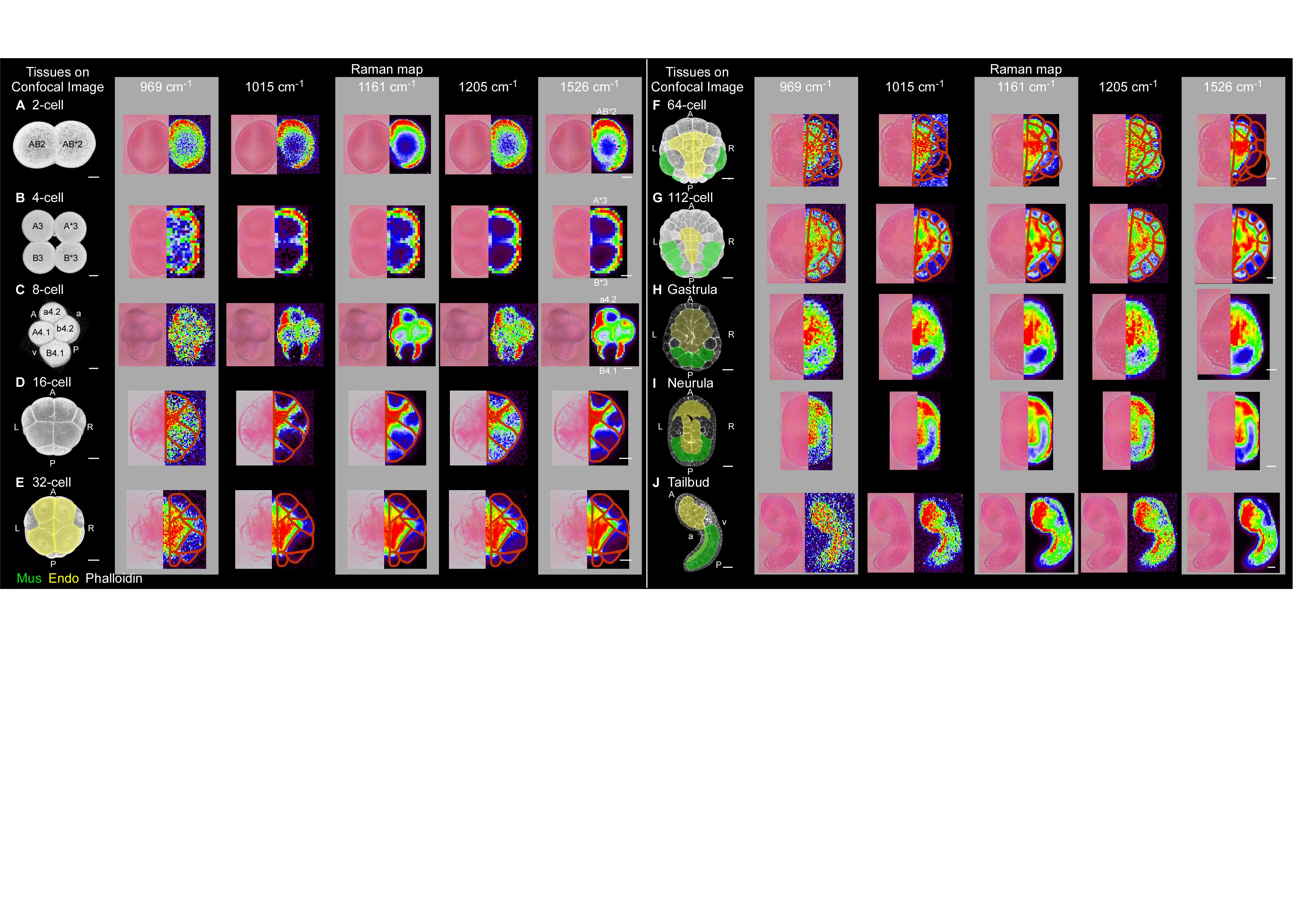

Supplement: Figure S3 — Raman maps of group II from 2-cell to tailbud stage embryos stained with CellMask. (A–J) (left) Confocal 3D (from 2-cell to 112-cell stages) and section images from dorsal (gastrula and neurula stages) or side views (tailbud stage) of C. intestinalis embryos stained with phalloidin (white). Images show the location of differentiated muscle (green) and endoderm (yellow) within the embryo. Right halves of Raman maps in a rainbow scale constructed from Raman bands at 969, 1015, 1161, 1205, and 1526 cm−1 are represented with bright-field image (left half) at each stage. These bands were categorized into group II according to the standard written in Results and Discussion. Embryos were stained with CellMask. Red line in Raman maps from 16-cell to 112-cell stage represents cell border presumed from the bright-field images and Raman maps of group III. Abbreviations: A, Anterior; P, Posterior; L, Left; R, Right; a, animal; v, vegetal; Mus, muscle; Endo, endoderm. Scale bar: 20 μm. (TIF) [file pone.0071739.s003.tif]

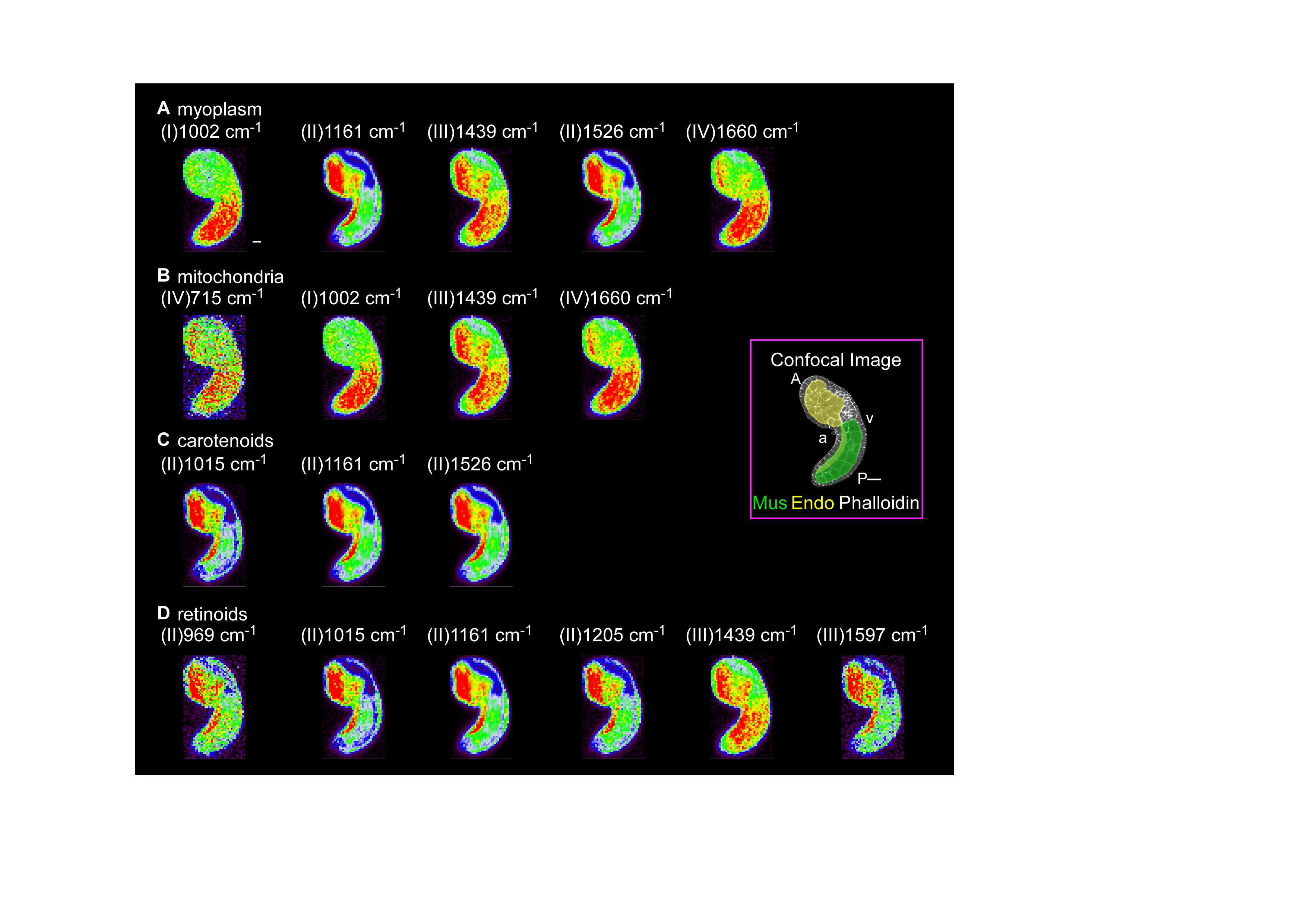

Supplement: Figure S4 — Raman maps constructed from major Raman bands of myoplasm, mitochondria, carotenoids and retinoids. Raman maps constructed from C. intestinalis embryos at the tailbud stage in the absence of CellMask. (A–D) Raman maps that represent Raman intensity at each embryonic region, depicted in rainbow scale. These maps were constructed from major Raman bands of myoplasm (A), mitochondria (B), carotenoids (C), and retinoids (D). Roman numerals (I–IV) represent the group into which each Raman band was classified. (Right panel) Confocal section image from side views of C. intestinalis embryo at the tailbud stage stained with phalloidin (white). Image shows the location of differentiated muscle (green) and endoderm (yellow) within the embryo. Abbreviations: A, Anterior; P, Posterior; a, animal; v, vegetal; Mus, muscle; Endo, endoderm. Scale bar: 20 μm. (TIF) [file pone.0071739.s004.tif]

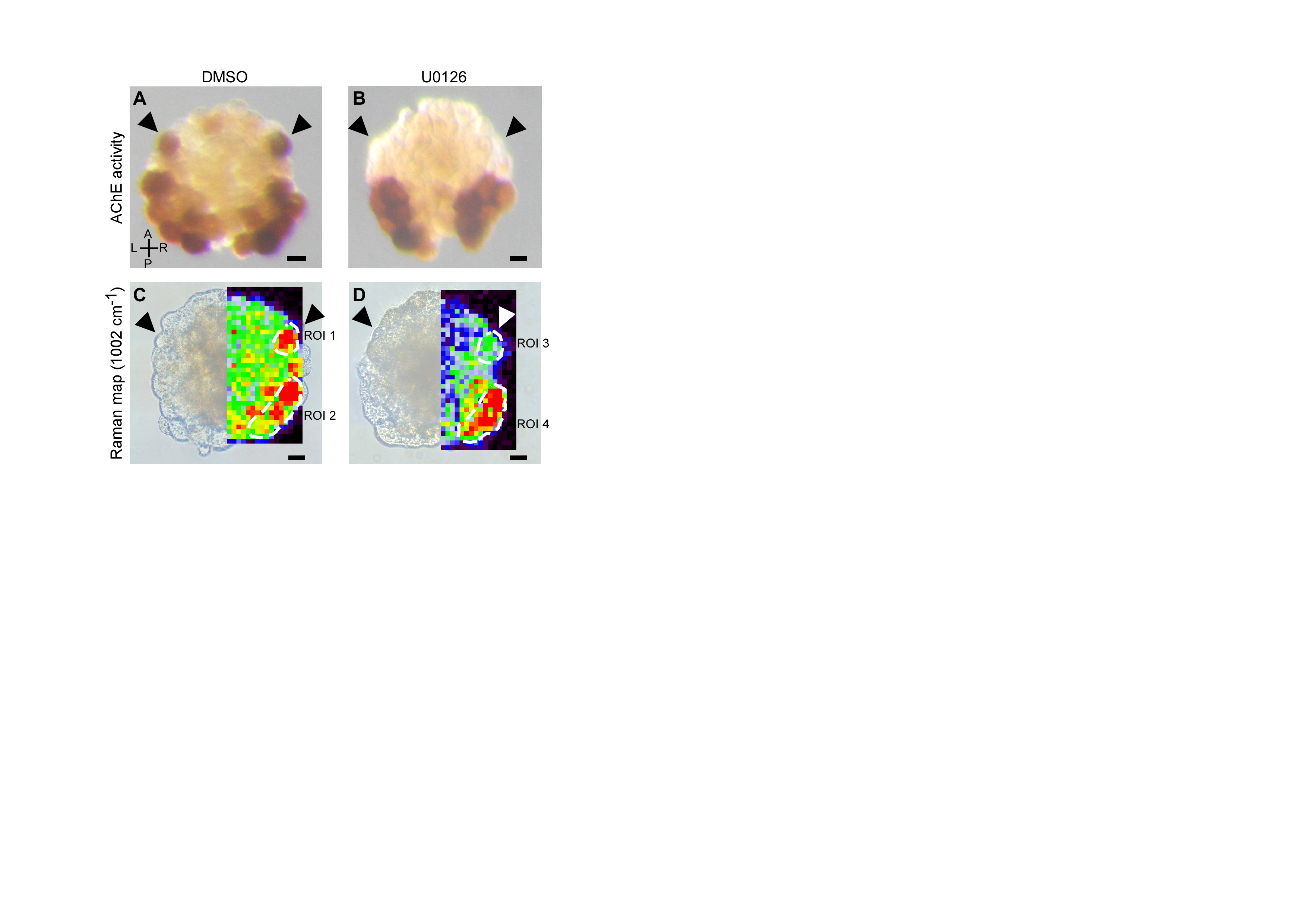

Supplement: Figure S5 — Verification of muscle differentiation of A8.16 blastomere by acetylcholinesterase (AChE) activity and Raman spectroscopy. (A–D) Muscle differentiation was verified by histochemical staining for AChE activity (A and B, brown) and Raman spectroscopy (C and D, red). Raman maps (C and D) were constructed based on the intensity of the single Raman band at the 1002 cm−1, and were represented in a rainbow scale. The scanning step size was 5.6 μm in both the x and y directions. Embryos were treated with DMSO (A and C) or U0126 (B and D) at the 8-cell stage, cytochalasin D at the 76-cell stage, and cultured until hatching stage (22 hpf). Arrowheads indicate A8.16 blastomeres. Inhibition of muscle differentiation of A8.16 blastomeres in U0126-treated embryos was detected by histochemical staining for AChE activity because brown products were not observed in A8.16 blastomeres (B, arrowheads). (C and D) Dotted white lines show region of interests (ROIs) encompassing A8.16 blastomere (ROI 1 and 3) and primary muscle region (ROI 2 and 4). Each ROI was used for the calculation of the intensity. Student's t-test result showed significant differences between the intensity of A8.16 blastomere and primary muscle region in U0126-treated embryo (n = 6, p<0.005) while DMSO-treated embryo showed no significant differences (n = 4, p>0.15). All embryos are in vegetal pole view. Abbreviations: A, Anterior; P, Posterior; L, Left; R, Right. Scale bar: 20 μm. (TIF) [file pone.0071739.s005.tif]
